# Headache and orofacial pain: A traffic-light prognosis-based management approach for the musculoskeletal practice

**DOI:** 10.3389/fneur.2023.1146427

**Published:** 2023-02-20

**Authors:** Tzvika Greenbaum, Alona Emodi-Perlman

**Affiliations:** ^1^Department of Physical Therapy, Faculty of Health Sciences, Recanati School for Community Health Professions, Ben-Gurion University of the Negev, Beer Sheva, Israel; ^2^Department of Oral Rehabilitation, The Maurice and Gabriela Goldschleger School of Dental Medicine, Sackler Faculty of Medicine, Tel Aviv University, Tel Aviv, Israel

**Keywords:** headache, orofacial pain, cervicogenic headache, temporomandibular joint, cervical spine, musculoskeletal pain, rehabilitation, physiotherapy

## Abstract

**Introduction:**

Headache (HA) is one of the most prevalent disabling conditions worldwide and is classified as either primary or secondary. Orofacial pain (OFP) is a frequent pain perceived in the face and/or the oral cavity and is generally distinct from a headache, according to anatomical definitions. Based on the up-to-date classification of the International Headache Society, out of more than 300 specific types of HA only two are directly attributed to the musculoskeletal system: The cervicogenic HA and HA attributed to temporomandibular disorders. Because patients with HA and/or OFP frequently seek help in the musculoskeletal practice, a clear and tailored prognosis-based classification system is required to achieve better clinical outcomes.

**Purpose:**

The aim of perspective article is to suggest a practical traffic-light prognosis-based classification system to improve the management of patients with HA and/or OFP in the musculoskeletal practice. This classification system is based on the best available scientific knowledge based on the unique set-up and clinical reasoning process of musculoskeletal practitioners.

**Implications:**

Implementation of this traffic-light classification system will improve clinical outcomes by helping practitioners invest their time in treating patients with significant involvement of the musculoskeletal system in their clinical presentation and avoid treating patients that are not likely to respond to a musculoskeletal based intervention. Furthermore, this framework incorporates medical screening for dangerous medical conditions, and profiling the psychosocial aspects of each patient; thus follows the biopsychosocial rehabilitation paradigm.

## 1. Headache and orofacial pain in the musculoskeletal practice

Headache (HA) is defined as “pain located in the head, above the orbitomeatal line and/or nuchal ridge” (1). According to the Global Burden of Disease (GBD) study, HA disorders are among the most prevalent and disabling conditions worldwide, with an estimated global prevalence of active HA disorder of 52.0% (95% CI 48.9–55.4) (2). According to the International Headache Society (IHS), HA is classified as either primary or secondary, based on its pathophysiological nature (1).

**Figure 1:**
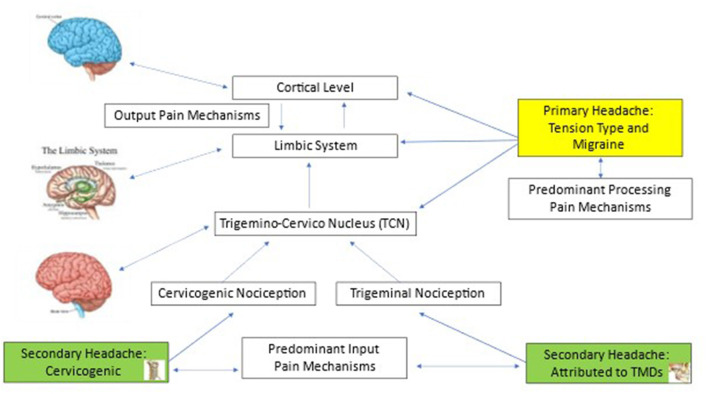
The neuroanatomical basis for primary and Musculoskeletal Headache & Orofacial Pain.

Primary HA is the most prevalent type of HA. It refers to an HA with an absence of a clear underlying causative pathology, trauma, or systemic disease to cause it (1). The most common primary HA is tension-type headache (TTH), with a current prevalence of 26% (95% CI 22.7–29.5), followed by migraine, with a current prevalence of 14% (95% CI 12.9–15.2) (2). Both TTH and migraine are more prevalent among women (TTH 22–34%; migraine 16–30%), especially during the fertility age (2). Primary HA is likely to become persistent and is defined as chronic daily primary HA when it occurs in a frequency of at least 15 episodes per month for the last 3 months (1, 3). Due to the convergence of sensory input from the upper cervical spine and the trigeminal nerve into the trigeminocervical nucleus of the brainstem, nociception from both the upper neck and the masticatory system has the potential to play a role in the neurophysiology of primary HA (Figure 1) (4, 5). However, although patients with migraine are very likely to complain about neck pain, it is shown that their cervical spine is not necessarily objectively impaired (6–8). Since primary HAs lack specific underlying pathology, they are considered a pain disorder, and as such, management approaches are multi-disciplinary and include among other treatment modalities, physiotherapy with some evidence to support it (9). The quality of evidence to support physiotherapy interventions for primary HA however is moderate for TTH (9, 10) and limited for migraine (9–12) and, thus, might be beneficial in combination with other therapies such as pharmacotherapy and cognitive behavioral therapy (CBT) to achieve better outcomes.

Secondary HA refers to an HA that is caused by a specific underlying medical condition, such as infection or homeostasis disorder (metabolic disturbance, e.g., hypoxia) (1). Secondary HA may present a diagnostic challenge as the symptomatology of different HA types such as headaches associated with sinusitis and/or infection and/or temporomandibular disorders (TMDs) often overlap (13, 14). The third edition of the International Classification of HA Disorders describes more than 300 distinct forms of secondary HAs; among them, two main forms of HA are of musculoskeletal (MSK) origin: cervicogenic HA (CGH) and an HA that is attributed to temporomandibular disorder (HA attributed to TMD) (1). The pathophysiology of both HAs is explained by the convergence of noxious stimuli from the upper cervical spine and the facial part of the cranium into the same neuroanatomical structure, trigeminocervical complex (Figure 1) (15, 16). Both forms of MSK HAs respond well to physiotherapy interventions such as manual and exercise therapies (17–19). Another two forms of HA that predominantly involve the MSK system are the acute and the persistent HAs attributed to whiplash (1), both considered as multi-system disorders in which the MSK system is the only component of the clinical presentation (20, 21). All other secondary forms of HA do not directly involve the MSK system. The management approaches for secondary HAs are based on the origin from where they arise, as such cervicogenic HA and HA that are attributed to TMD are successfully managed by MSK clinicians (18, 21).

Orofacial pain (OFP) is defined as “a frequent form of pain perceived in the face and/or the oral cavity” (22) and is generally distinct from HA based on anatomic definitions (23). While HA landmarks are above the orbitomeatal and/or nuchal ridge, OFP is anatomically described as pain occurring mainly or exclusively under the orbitomeatal line, anterior to the pinnae, and above the neck (1, 23). OFP is common with a prevalence of around 25% (23, 24). OFP is known to induce a significant reduction in the quality of life, sleep disturbances, and disability levels (23, 25). Pain-related TMD is the leading diagnosis of OFP, with a prevalence of 10–15% in adults (26), followed by primary HA that is expressed in the facial region, common ear–nose–throat pathologies, and dental disorders (23). Interestingly, CGH is very likely to be expressed unilaterally in the facial area due to its underlying trigeminocervical pathophysiology (16, 27). Therefore, the two unique MSK headaches that were debrided before (cervicogenic and attributed to TMD) may be presented and considered as forms of OFP as well.

Chronic primary HA and OFP share an association with mental disorders such as anxiety and depression (28). The association of migraine HA to both depressive symptoms (24.9%) and anxiety disorder (20.5%) is higher than that of TTH (12.6 and 10.2%, respectively) (29). It is well-supported that both depression and anxiety disorders act risky, perpetuating contributing factors in the clinical presentation of patients with primary HA and, therefore, must be taken into consideration in their assessment and management (28).

## 2. Screening and classifying HA/OFP in the MSK practice: The traffic light approach

The relatively high prevalence of both TMD (30) and CGH (31) requires MSK clinicians to carefully monitor and assess patients with complaints of HA/OFP. The assessment should address and answer four main questions:

- May the presented HA/OFP be secondary to a dangerous medical condition?
- Is the presented HA/OFP secondary to a specific MSK disorder (CGH and/or TMDs)?
- If primary HA is presented, what is the expected response to MSK rehabilitation?
- How severe is the mental distress associated with HA/OFP?

**Figure 2:**
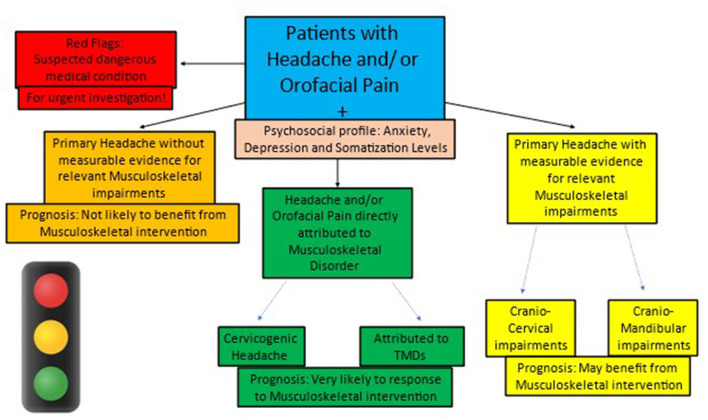
The traffic light classification of Headache & Orofacial Pain in the Musculoskeletal practice.

To assist clinicians in organizing their clinical reasoning process and answering these critical questions, a practical “traffic light” approach is suggested (Figure 2). According to this approach, all patients with HA/OFP can be classified into one of the four traffic light colors that describe both their condition and their prognosis: green (MSK condition, very likely to respond), yellow (non-MSK condition, may respond), orange (non-MSK condition, not likely to respond), and red (non-MSK, dangerous medical condition).

### 2.1. The red light: Will the presented HA/OFP be secondary to a dangerous medical condition?

Differentiating dangerous HA/OFP from benign or low-risk HA/OFP is an important and challenging task as symptoms may often overlap (32). Therefore, patients with secondary HA should be carefully evaluated to exclude the possibility of an underlying dangerous medical condition requiring fast and accurate medical management. While screening patients with HA/OFP for dangerous medical conditions, three main subgroups should be considered: acute infections (such as meningitis), arteriovenous pathologies (such as intra-cranial hemorrhage), and oncological pathologies (such as mass lesions) (32). The MSK clinician should carefully screen the patients with HA/OFP for each of the three subgroups of dangerous medical conditions and classify any suspected patient as “red light.” The most common red flag symptoms to consider are the first occurrence or worst headache ever experienced by the patient and focal neurological signs and headache that is triggered by a cough or exertion (32, 33). Patients that are classified as “red light” in the MSK practice should be referred as soon as possible to a relevant medical doctor.

### 2.2. The green light: Is the presented HA/OFP secondary to a specific MSK disorder (CGH and/or TMDs)?

Cervicogenic HA is defined as a “Headache caused by a disorder of the cervical spine and its bony component, disc and/or soft tissue elements, usually but not invariably accompanied by neck pain” (1). When combining the diagnostic criteria of the “International HA Society” (“International Headache Society,” n.d.) and the “Cervicogenic Headache International Study Group,” (34–37) patients with CGH are characterized by the following features: (1) The clinical and/or imaging evidence of neck disorder or lesion is known as able to cause HA; (2) HA has developed and/or improved close to the onset or improvement of cervical spine disorder or lesion; (3) The cervical range of motion is reduced, and HA is provoked/eased by neck maneuvers; (4) The persistent unilaterality or side dominancy of the HA without side shift; and (5) Headache is abolished following the diagnostic blockade of a cervical structure or its nerve supply.

Cervicogenic HA is usually presented with three main objective clinical features that allow the MSK clinicians to differentiate it from other forms of HA: (1) Impaired general neck mobility (38, 39), (2) Impaired selective upper neck mobility (40–43), and (3) Impaired cranio-cervical muscular function (44–47). While the impaired general neck mobility is clearly assessed by active physiological neck movements (39), the impaired upper neck mobility is clearly assessed by the cervical flexion-rotation test (FRT) (41, 48–50) that assesses the passive rotatory mobility of the upper cervical spine. Another valid way to assess the mobility of the upper neck is the manual segmental assessment of each one of the upper three neck motion segments, performed by well-trained manual therapists (38). Additional cervical spine impairment that is associated with CGH and not with primary HA is the impaired muscular performance of the cranio-cervical flexors (44, 46). This muscle group is assessed by two different clinical tests: (1) The cranio-cervical flexion test (18) and (2) The neck flexors endurance test (51). While the former better assesses the recruitment pattern of the flexors, the latter test better demonstrates its strength and endurance of it; hence, the combination of the two is recommended.

Combining the three clinical components (general neck mobility, specific upper neck mobility, and muscular performance of the cervical flexors) provides a valid and reliable cluster to differentiate patients with CGH from other patients with HA, allowing them to establish their excellent prognosis with physical rehabilitation (green light).

A headache attributed to TMD is defined as a “Headache caused by a disorder involving structures in the temporomandibular region.” (1). According to the diagnostic criteria for TMD (DC/TMD) (52), this specific type of HA is one of four specific pains related to TMD diagnoses together with “local myalgia,” “myofascial pain with referral,” and “arthralgia.” To classify the HA as attributed to TMD and differentiate it from other forms of TMDs and/or HA, it should have the following conditions: (1) Located in the temple area and must be influenced by jaw movements, function (for example chewing), and/or parafunction; (2) During the clinical examination of the patient (according to the AXIS I DC/TMD protocol) with a familiar pain, resembling the HA complaint, should be provoked with two tests such as the palpation of the temporalis muscle or following jaw movements (1, 52); and (3) Headache not better accounted for by another HA diagnosis. It is important to note that pain-related TMDs are highly associated with other forms of both primary HA (24, 53) and CGH (54–56); therefore, a concurrent diagnosis is very likely to co-occur or overlap.

### 2.3. The yellow and orange lights: If primary HA is presented, what is the expected response to MSK rehabilitation?

Statistically, the two most common differential diagnoses for MSK HA are the most common primary HA types: TTH and migraine. Additionally, some pieces of evidence support the upper neck involvement in the clinical presentations of both TTH (42, 57, 58) and migraine (8, 59). Interestingly, in migraine, cervical MSK findings may be real or apparent due to hypersensitivity which may lead to subjective neck complaints without objective neck impairments (6, 60, 61) and, therefore, should be carefully checked. Importantly, the current literature suggests very high co-morbidity rates of both TTH and migraine HA in patients with pain-related TMDs (62) with some evidence of the etiological relationship (53, 63).

In the assessment of patients with diagnosed primary HA, the main challenge of the MSK practitioner is to identify those who present significant objective and measurable MSK impairments (cranio-cervical and/or cranio-mandibular) in order to justify MSK interventions and expect a positive prognosis. These impairments should mainly include a decrease in pain-free active physiological neck/jaw movements and/or reduced upper neck pain-free mobility and/or the jaw and impaired muscular performance of the cervical and/or jaw musculature. In such a scenario, the patient is classified as “yellow light” since a significant improvement is expected when applying MSK interventions.

In case the primary patient with HA does not present significant objective measurable MSK impairments (cranio-cervical and/or cranio-mandibular) justifying MSK interventions, there is **NO** rationale to expect a positive prognosis other than the placebo effect. Therefore, these patients should be classified as “orange light” and be referred to other non-MSK-based interventions.

### 2.4. Psychosocial screening: How severe is the mental distress associated with the HA/OFP?

The DC/TMD Axis II (52) includes a thorough assessment of the psychosocial status of the patient with OFP. The component includes the assessment of anxiety, depression, and somatization levels using several valid and reliable self-report questionnaires. Three of these questionnaires may be used in the assessment of patients with HA/OFP in the MSK practice. The first one assesses anxiety levels with seven questions (GAD7) (64), the second assesses depressive levels with nine questions (PHQ9) (65), and the third assesses somatization levels with 15 questions (PHQ15) (66). A short version to assess the psychosocial aspects of patients with HA/OFP with four questions is PHQ4 (67). The combination of these first three scales may give a solid basis to evaluate the degree of mental distress and burden of the patients with HA/OFP. The effect on the quality of life of the patient should then be taken into consideration as it will influence both management strategies and the prognosis.

## 3. Management and prognosis of patients with HA/OFP in MSK practice

### 3.1. Management and prognosis of CGH

Based on its pathophysiology, the important MSK structures that are potential sources for CGH are innervated by the three upper spinal cervical segments. Therefore, the manual techniques relevant to the management of patients with CGH are tailored to the 0CC-C1-C2-C3 segments to de-sensitize the trigeminocervical nucleus (TCN) by reducing the nociception from the peripheral structure and facilitating the descending inhibitory pathways (68) (Figure 1). This tailored upper neck segmental manual therapy has been proven to significantly improve the condition of most patients with CGH, both in the short and long terms (17, 69).

In addition to segmental manual therapy, the upper neck musculature is also likely to play a major role both as the pain generator (42) and as a source of motor dysfunction (21, 46, 47). Based on clinical observation, the muscles that are mostly considered to be involved both as pain generators and movement restrictors in CGH are the suboccipital, splenius capitis, sternocleidomastoid, and upper trapezius. Treating these muscles with manual techniques and dry needling demonstrated a significantly improved prognosis in patients with CGH (70). To improve the motor function of the deep cervical flexors in patients with CGH, a specific multi-phase exercise program is required starting with a recruitment phase, followed by an endurance phase, and ending with the power phase, which is more relevant to the athletic population (21).

### 3.2. Management and prognosis of HA attributed to TMD

The main source of pain arises from the masticatory muscles, especially the three mouth closers: masseter, temporalis, and medial pterygoid muscles. Therefore, the management of these patients should be focused in the short term on the myofascial pain technique (manual therapy and dry needling) (18, 71) and addressing the contributing factors for muscular pain in the longer term (especially the reduction of awake bruxism muscle behavior and/or other oral parafunction that may act as risk factors) (72). Very commonly the upper neck plays a role in the presentation of pain-related TMDs (54–56) and, therefore, should be considered a major potential factor in the management of patients with HA attributed to TMDs. Another important factor for patients with TMDs in general and especially with pain-related TMDs is the psychosocial component (30). It was demonstrated that patients with referred facial pain (and those with HA attributed to TMDs) are more likely to have higher levels of anxiety and depression compared to patients with local myalgia (73). The MSK clinician should be aware, monitor, assess, and apply up-to-date chronic pain management strategies including pacing and psychoeducation to control the aggravating influence of the psychosocial component of pain (74, 75).

### 3.3. Management and prognosis of primary HA

As explained previously in the classification section, patients with primary HA may benefit from MSK interventions only if significant measurable cervico-cranio-mandibular impairments are present (“Yellow” light). It is also important to notice that while some pieces of evidence support the effectiveness of MSK interventions for TTH HA, the quality of evidence to support it for migraine is very limited (6, 9, 10, 60, 61). The management of these patients should be tailored to the presented measurable cranio-cervical and/or cranio-mandibular impairments, and the prognosis of patients with migraine is unclear. Therefore, the key factor in the management of “Yellow” light patients is the careful monitoring of their response to the MSK intervention. Primary HA episodes are well-defined by four measurable clinical parameters: intensity, duration, frequency, and medication use. The MSK clinician should apply the intervention based on the measurable clinical MSK findings (mobility, strength, and endurance) and correlate it with the trend of the measurable HA episode parameters. In the case of associated improvement of both MSK measurable parameters and HA episodes, the justification to continue therapy exists and should continue until a plateau is reached. In the case of dissociated trends, therapy should end, and the patient is likely to be categorized as “Orange.”

## 4. Summary and clinical implications

Patients with HA commonly present MSK impairments and therefore are treated by MSK clinicians, with variable outcomes. To maximize the positive response and outcome, a new practical prognosis-based framework is suggested in this perspective article. According to this framework, patients with HA should be categorized into four traffic-light colors: red, green, yellow, and orange. The meaning of each color is the expected prognosis of the patient by receiving the relevant MSK intervention (cranio-cervical and/or cranio-mandibular). “Red” patients are those who present signs and symptoms of dangerous HA and therefore should be referred immediately for further emergency medical evaluation and management. “Green” patients are those who present pure MSK HA (CGH and HA attributed to the TMDs) and, therefore, should receive the appropriate MSK management with an excellent prognosis. “Yellow” patients presenting primary HA with significant measurable MSK impairments should be treated by the MSK clinician with a certain degree of an expected positive prognosis. Finally, “Orange” patients are those with primary HA without measurable MSK impairments and, therefore, are not likely to benefit from MSK interventions.

## Data availability statement

The raw data supporting the conclusions of this article will be made available by the authors, without undue reservation.

## Author contributions

All authors fully cooperated in developing the presented clinical concept, organizing it into a clinical perspective, and writing it as an article.

## References

[1] Olesen J. Headache classification committee of the International Headache Society (IHS) the international classification of headache disorders, 3rd edition. Cephalalgia. (2018) 38:1–211. 10.1177/0333102417738202 29368949

[2] Stovner LJ Hagen K Linde M Steiner TJ. The global prevalence of headache: An update, with analysis of the influences of methodological factors on prevalence estimates. J Headache Pain. (2022) 23:34. 10.1186/s10194-022-01402-2 35410119 PMC9004186

[3] Murphy C Hameed S. Chronic Headaches. StatPearls. (2022). Available online at: https://www.ncbi.nlm.nih.gov/books/NBK559083/ (accessed January 24, 2023).

[4] Maurice B Vincent MDP. Cervicogenic headache: The neck is a generator: Con. Headache. (2010) 50:699–705. 10.1111/j.1526-4610.2010.01648.x 20456157

[5] Marfurt CF Rajchert DM. Trigeminal primary afferent projections to “non-trigeminal” areas of the rat central nervous system. J Comp Neurol. (1991) 303:489–511. 10.1002/cne.903030313 1706735

[6] Liang Z Thomas L Jull G Minto J Zareie H Treleaven J. Neck pain associated with migraine does not necessarily reflect cervical musculoskeletal dysfunction. Headache. (2021) 61:882–94. 10.1111/head.14136 34214181

[7] Liang Z Thomas L Jull G Treleaven J. The neck disability index reflects allodynia and headache disability but not cervical musculoskeletal dysfunction in migraine. Phys Ther. (2022) 102:1–14. 10.1093/ptj/pzac027 35230421 PMC9156011

[8] Di Antonio S Arendt-Nielsen L Ponzano M Bovis F Torelli P Finocchi C. Cervical musculoskeletal impairments in the 4 phases of the migraine cycle in episodic migraine patients. Cephalalgia. (2022) 42:827–45. 10.1177/03331024221082506 35332826

[9] Varangot-Reille C Suso-Martí L Dubuis V Cuenca-Martínez F Blanco-Díaz M Salar-Andreu C. Exercise and manual therapy for the treatment of primary headache: An umbrella and mapping review. Phys Ther. (2022) 102:pzab308. 10.1093/ptj/pzab308 35084039

[10] Varangot-Reille C Suso-Martí L Romero-Palau M Suárez-Pastor P Cuenca-Martínez F. Effects of different therapeutic exercise modalities on migraine or tension-type headache: A systematic review and meta-analysis with a replicability analysis. J Pain. (2021) 12:3. 10.1016/j.jpain.2021.12.003 34929374

[11] Beier D Callesen HE Carlsen LN Birkefoss K Tómasdóttir H Wurtzen H. Manual joint mobilisation techniques, supervised physical activity, psychological treatment, acupuncture and patient education in migraine treatment. A systematic review and meta-analysis. Cephalalgia. (2022) 42:63–72. 10.1177/03331024211034489 34404258

[12] Benatto MT Florencio LL Bragatto MM Dach F Fernández-de-las-Peñas C Bevilaqua-Grossi D. Neck-specific strengthening exercise compared with placebo sham ultrasound in patients with migraine: A randomized controlled trial. BMC Neurol. (2022) 22:1–13. 10.1186/s12883-022-02650-0 35366822 PMC8976325

[13] Wieckiewicz M Grychowska N Nahajowski M Hnitecka S Kempiak K Charemska K. Prevalence and overlaps of headaches and pain-related temporomandibular disorders among the polish urban population. J Oral Facial Pain Headache. (2020) 34:31–9. 10.11607/ofph.2386 31465030

[14] Straburzyński M Nowaczewska M Budrewicz S Waliszewska-Prosół M. COVID-19-related headache and sinonasal inflammation: A longitudinal study analysing the role of acute rhinosinusitis and ICHD-3 classification difficulties in SARS-CoV-2 infection. Cephalalgia. (2022) 42:218–28. 10.1177/03331024211040753 34541916 PMC8988454

[15] Goadsby P Bartch T. The anatomy and physiology of the trigeminocervical complex. In: C Fernandez-de-las-penas, L Ardent-Nielsen, R Gerwin, editors, Tension Type and Cervicogenic Headache: Pathophysiology, Diagnosis and Management. London: Jones and Bartlett Publishers (2010). p. 109–16.

[16] Bartsch T Goadsby PJ. Increased responses in trigeminocervical nociceptive neurons to cervical input after stimulation of the dura mater. Brain. (2003) 126:1801–13. 10.1093/brain/awg190 12821523

[17] Jull G Trott P Potter H Zito G Niere K Shirley D. A randomized controlled trial of exercise and manipulative therapy for cervicogenic headache. Spine. (2002) 27:1835–42. 10.1097/00007632-200209010-00004 12221344

[18] Calixtre LB Moreira RFC Franchini GH Alburquerque-Sendín F Oliveira AB. Manual therapy for the management of pain and limited range of motion in subjects with signs and symptoms of temporomandibular disorder: A systematic review of randomised controlled trials. J Oral Rehabil. (2015) 42:847–61. 10.1111/joor.12321 26059857

[19] Luedtke K Allers A Schulte LH May A. Efficacy of interventions used by physiotherapists for patients with headache and migraine-systematic review and meta-analysis. Cephalalgia. (2016) 36:474–92. 10.1177/0333102415597889 26229071

[20] Godek P. Whiplash injuries. Current state of knowledge. Ortop Traumatol Rehabil. (2020) 22:293–302. 10.5604/01.3001.0014.4210 33568566

[21] Jull G Sterling M Falla D Treleaven J O'Leary S. Principles of management of cervical disorders. In: G Jull, M Sterling, D Falla, J, Treleaven S, O'Leary, editors, Whiplash, Headache and Neck Pain. Elsevier Health Sciences (2008). p. 189–216. 10.1016/B978-0-443-10047-5.50017-5

[22] Peck CC Goulet JP Lobbezoo F Schiffman EL Alstergren P Anderson GC. Expanding the taxonomy of the diagnostic criteria for temporomandibular disorders. J Oral Rehabil. (2014) 41:2–23. 10.1111/joor.12132 24443898 PMC4520529

[23] Ananthan S Benoliel R. Chronic orofacial pain. J Neural Transm. (2020) 127:575–88. 10.1007/s00702-020-02157-3 32130516

[24] De Melo PC Lins Aroucha JMCN Arnaud M De Souza Lima MG Gomes SGF Ximenes R. Prevalence of TMD and level of chronic pain in a group of Brazilian adolescents. PLoS ONE. (2019) 14:e0205874. 10.1371/journal.pone.0205874 30735506 PMC6368276

[25] Benoliel R Zini A Zakuto A Slutzky H Haviv Y Sharav Y. Subjective sleep quality in temporomandibular disorder patients and association with disease characteristics and oral health-related quality of life. J Oral Facial Pain Headache. (2017) 31:313–22. 10.11607/ofph.1824 28973048

[26] List T Jensen RH. Temporomandibular disorders: Old ideas and new concepts. Cephalalgia. (2017) 37:692–704. 10.1177/0333102416686302 28068790

[27] Biondi DM. Cervicogenic headache: A review of diagnostic and treatment strategies. J Am Osteopath Assoc. (2005) 105:16S–22S. 10.7556/jaoa.2005.20010 15928349

[28] Caponnetto V Deodato M Robotti M Koutsokera M Pozzilli V Galati C. Comorbidities of primary headache disorders: A literature review with meta-analysis. J Headache Pain. (2021) 22:1281. 10.1186/s10194-021-01281-z 34261435 PMC8278743

[29] Porst M Wengler A Leddin J Neuhauser H Katsarava Z von der Lippe E. Migraine and tension-type headache in Germany. Prevalence and disease severity from the BURDEN 2020 Burden of Disease Study. J Heal Monit. (2020) 5:2–24. 10.25646/6990.2 35146296 PMC8734075

[30] Sonia S Ohrbach R. Definition, epidemiology and etiology of painful temporomandibular disorders. In: C Fernandez-De-La-Penas, J Mesa-Jimenez, editors, Temporomandibular Disorders. Edenborough: Handspring Publishing (2018). p. 3–22.

[31] Knackstedt H Bansevicius D Aaseth K BerlingGrande R Lundqvist C Russell MB. Cervicogenic headache in the general population: The Akershus study of chronic headache. Cephalalgia. (2010) 30:1468–76. 10.1177/0333102410368442 20974607

[32] Hainer BL Matheson EM. Approach to acute headache in adults. Am Fam Physician. (2013) 87:682–7. 23939446

[33] Do TP Remmers A Schytz HW Schankin C Nelson SE Obermann M. Red and orange flags for secondary headaches in clinical practice: SNNOOP10 list. Neurology. (2019) 92:134. 10.1212/WNL.0000000000006697 30587518 PMC6340385

[34] Sjaastad O Fredriksen TA Pfaffenrath V. Cervicogenic headache: diagnostic criteria. The Cervicogenic Headache International Study Group. Headache. (1998) 38:442–5. 10.1046/j.1526-4610.1998.3806442.x 9664748

[35] Sjaastad O. Cervicogenic headache: Comparison with migraine without aura; Vågå study. Cephalalgia. (2008) 28:18–20. 10.1111/j.1468-2982.2008.01610.x 18494988

[36] Van Suijlekom JA De Vet HCW Van den Berg SGM Weber WEJ. Interobserver reliability of diagnostic criteria for cervicogenic headache. Cephalalgia. (1999) 19:817–23. 10.1046/j.1468-2982.1999.1909817.x 10595292

[37] Sjaastad O. Reliability of cervicogenic headache diagnosis. Cephalalgia. (1999) 19:767–8. 10.1046/j.1468-2982.1999.19097657.x 10595284

[38] Getsoian SL Gulati SM Okpareke I Nee RJ Jull GA. Validation of a clinical examination to differentiate a cervicogenic source of headache: A diagnostic prediction model using controlled diagnostic blocks. Br Med J Open. (2020) 10:1–9. 10.1136/bmjopen-2019-035245 32376753 PMC7223143

[39] Amiri M Jull G Bullock-Saxton J Darnell R Lander C. Cervical musculoskeletal impairment in frequent intermittent headache. Part 2: Subjects with concurrent headache types. Cephalalgia. (2007) 27:891–8. 10.1111/j.1468-2982.2007.01346.x 17608813

[40] Ogince M Hall T Robinson K Blackmore AM. The diagnostic validity of the cervical flexion-rotation test in C1/2-related cervicogenic headache. Man Ther. (2007) 12:256–62. 10.1016/j.math.2006.06.016 17112768

[41] Satpute K Nalband S Hall T. The C0-C2 axial rotation test: Normal values, intra- and inter-rater reliability and correlation with the flexion rotation test in normal subjects. J Man Manip Ther. (2019) 27:1533195. 10.1080/10669817.2018.1533195 30935342 PMC6484503

[42] Jull G Hall T. Cervical musculoskeletal dysfunction in headache: How should it be defined? Musculoskelet Sci Pract. (2018) 38:148–50. 10.1016/j.msksp.2018.09.012 30270129

[43] Hall TM Robinson KW Fujinawa O Akasaka K Pyne EA. Intertester reliability and diagnostic validity of the cervical flexion-rotation test. J Manipulative Physiol Ther. (2008) 31:293–300. 10.1016/j.jmpt.2008.03.012 18486750

[44] Anarte-Lazo E Carvalho GF Schwarz A Luedtke K Falla D. Differentiating migraine, cervicogenic headache and asymptomatic individuals based on physical examination findings: A systematic review and meta-analysis. BMC Musculoskelet Disord. (2021) 22:4595. 10.1186/s12891-021-04595-w 34479514 PMC8417979

[45] Rubio-Ochoa J Benítez-Martínez J Lluch E Santacruz-Zaragozá S Gómez-Contreras P Cook CE. Physical examination tests for screening and diagnosis of cervicogenic headache: A systematic review. Man Ther. (2016) 21:35–40. 10.1016/j.math.2015.09.008 26423982

[46] O'leary S Falla D Elliott JM Jull G. Muscle dysfunction in cervical spine pain: Implications for assessment and management. J Orthopaed Sports Phys Ther. (2009) 2009:324–33. 10.2519/jospt.2009.2872 19411767

[47] Jull GA O'Leary SP Falla DL. Clinical assessment of the deep cervical flexor muscles: The craniocervical flexion test. J Manipulative Physiol Ther. (2008) 31:525–33. 10.1016/j.jmpt.2008.08.003 18804003

[48] Takasaki H Hall T Oshiro S Kaneko S Ikemoto Y Jull G. Normal kinematics of the upper cervical spine during the Flexion-Rotation Test—*In vivo* measurements using magnetic resonance imaging. Man Ther. (2011) 16:167–71. 10.1016/j.math.2010.10.002 21055995

[49] Hall TM Briffa K Hopper D Robinson KW. The relationship between cervicogenic headache and impairment determined by the flexion-rotation test. J Manipulative Physiol Ther. (2010) 33:666–71. 10.1016/j.jmpt.2010.09.002 21109057

[50] Hall TM Briffa K Hopper D Robinson K. Comparative analysis and diagnostic accuracy of the cervical flexion-rotation test. J Headache Pain. (2010) 11:391–7. 10.1007/s10194-010-0222-3 20508964 PMC3452271

[51] Edmondston SJ Wallumrød ME MacLéid F Kvamme LS Joebges S Brabham GC. Reliability of isometric muscle endurance tests in subjects with postural neck pain. J Manipulative Physiol Ther. (2008) 31:348–54. 10.1016/j.jmpt.2008.04.010 18558277

[52] Schiffman E Ohrbach R Truelove E Look J Anderson G Goulet J-P. Diagnostic criteria for temporomandibular disorders (DC/TMD) for clinical and research applications: Recommendations of the international RDC/TMD consortium network^*^ and orofacial pain special interest group. J Oral Facial Pain Headache. (2014) 28:6–27. 10.11607/jop.1151 24482784 PMC4478082

[53] Costa YM Conti PCR de Faria FAC Bonjardim LR. Temporomandibular disorders and painful comorbidities: Clinical association and underlying mechanisms. Oral Surg Oral Med Oral Pathol Oral Radiol. (2017) 123:5. 10.1016/j.oooo.2016.12.005 28153123

[54] Greenbaum T Dvir Z Reiter S Winocur E. Cervical flexion-rotation test and physiological range of motion—A comparative study of patients with myogenic temporomandibular disorder versus healthy subjects. Musculoskelet Sci Pract. (2017) 27:10. 10.1016/j.msksp.2016.11.010 28637604

[55] Greenbaum T Dvir Z Emodi-Perelmam A Reiter S Rubin P Winocur E. Relationship between specific temporomandibular disorders and impaired upper neck performance. Eur J Oral Sci. (2020) 128:292–8. 10.1111/eos.12718 32627243

[56] Greenbaum T Dvir Z Emodi-Perlman A Reiter S Rubin P Winocur E. The association between specific temporomandibular disorders and cervicogenic headache. Musculoskelet Sci Pract. (2021) 52:102321. 10.1016/j.msksp.2021.102321 33482538

[57] Castien R Duineveld M Maaskant J De Hertogh W Scholten-Peeters G. Pericranial total tenderness score in patients with tension-type headache and migraine. A systematic review and meta-analysis. Pain Physician. (2021) 24:E1177–89. 34793636

[58] Watson DH Drummond PD. Head pain referral during examination of the neck in migraine and tension-type headache. Headache. (2012) 52:1226–35. 10.1111/j.1526-4610.2012.02169.x 22607581

[59] Di Antonio S Castaldo M Ponzano M Bovis F Hugo Villafañe J Torelli P. Trigeminal and cervical sensitization during the four phases of the migraine cycle in patients with episodic migraine. Headache. (2022) 62:176–90. 10.1111/head.14261 35122434

[60] Liang Z Galea O Thomas L Jull G Treleaven J. Cervical musculoskeletal impairments in migraine and tension type headache: A systematic review and meta-analysis. Musculoskelet Sci Pract. (2019) 42:7. 10.1016/j.msksp.2019.04.007 31054485

[61] Liang Z Thomas L Jull G Treleaven J. The temporal behaviour of migraine related neck pain does not inform on the origin of neck pain: An observational study. Musculoskelet Sci Pract. (2022) 58:102522. 10.1016/j.msksp.2022.102522 35121244

[62] Réus JC Polmann H Souza BDM Flores-Mir C Gonçalves DAG de Queiroz LP. Association between primary headaches and temporomandibular disorders: A systematic review and meta-analysis. J Am Dent Assoc. (2022) 153:120–31.e6. 10.1016/j.adaj.2021.07.021 34649707

[63] Cruz D Monteiro F Paço M Vaz-Silva M Lemos C Alves-Ferreira M. Genetic overlap between temporomandibular disorders and primary headaches: A systematic review. Jpn Dent Sci Rev. (2022) 58:69–88. 10.1016/j.jdsr.2022.02.002 35242249 PMC8881721

[64] Tiirikainen K Haravuori H Ranta K Kaltiala-Heino R Marttunen M. Psychometric properties of the 7-item Generalized Anxiety Disorder Scale (GAD-7) in a large representative sample of Finnish adolescents. Psychiatry Res. (2019) 272:30–5. 10.1016/j.psychres.2018.12.004 30579178

[65] Manea L Gilbody S McMillan D. A diagnostic meta-analysis of the Patient Health Questionnaire-9 (PHQ-9) algorithm scoring method as a screen for depression. Gen Hosp Psychiatry. (2015) 37:67–75. 10.1016/j.genhosppsych.2014.09.009 25439733

[66] Van Ravesteijn H Wittkampf K Lucassen P Van De Lisdonk E Van Den Hoogen H Van Weert H. Detecting somatoform disorders in primary care with the PHQ-15. Ann Fam Med. (2009) 7:232–8. 10.1370/afm.985 19433840 PMC2682971

[67] Kroenke K Spitzer RL Williams JBW Löwe B. An ultra-brief screening scale for anxiety and depression: the PHQ-4. Psychosomatics. (2009) 50:613–21. 10.1016/S0033-3182(09)70864-3 19996233

[68] Bialosky J Bishop M Price D Robinson M George S. Mechanisms of manual therapy. Mech Man Ther. (2008) 14:103. 10.1016/j.math.2008.09.001 19027342 PMC2775050

[69] Racicki S Gerwin S Diclaudio S Reinmann S Donaldson M. Conservative physical therapy management for the treatment of cervicogenic headache: A systematic review. J Man Manip Ther. (2013) 21:113–24. 10.1179/2042618612Y.0000000025 24421621 PMC3649358

[70] Pourahmadi M Dommerholt J Fernández-De-Las-Peñas C Koes BW Mohseni-Bandpei MA Mansournia MA. Dry needling for the treatment of tension-type, cervicogenic, or migraine headaches: A systematic review and meta-analysis. Phys Ther. (2021) 101:pazb068. 10.1093/ptj/pzab068 33609358

[71] Shaffer SM Brismée JM Sizer PS Courtney CA. Temporomandibular disorders. Part 2: Conservative management. J Man Manip Ther. (2014) 22:13–23. 10.1179/2042618613Y.0000000061 24976744 PMC4062348

[72] Story WP Durham J Al-Baghdadi M Steele J Araujo-Soares V. Self-management in temporomandibular disorders: A systematic review of behavioural components. J Oral Rehabil. (2016) 43:759–70. 10.1111/joor.12422 27487973

[73] Winocur-Arias O Friedman-Rubin P Abu Ras K Lockerman L Emodi-Perlman A Greenbaum T. Local myalgia compared to myofascial pain with referral according to the DC/TMD: Axis I and II results. BMC Oral Health. (2022) 22:2048. 10.1186/s12903-022-02048-x 35120492 PMC8815134

[74] Galvez-Sánchez CM Montoro CI Moreno-Padilla M Reyes Del Paso GA de la Coba P. Effectiveness of acceptance and commitment therapy in central pain sensitization syndromes: A systematic review. J Clin Med. (2021) 10:122706. 10.3390/jcm10122706 34205244 PMC8235706

[75] Sánchez-Gutiérrez C Gil-García E Rivera-Sequeiros A López-Millán JM. Effectiveness of telemedicine psychoeducational interventions for adults with non-oncological chronic disease: A systematic review. J Adv Nurs. (2022) 78:1267–80. 10.1111/jan.15151 35075690

